# Developing a typology of interventions to support doctors’ mental health and wellbeing

**DOI:** 10.1186/s12913-024-10884-6

**Published:** 2024-05-03

**Authors:** Alison Pearson, Daniele Carrieri, Anna Melvin, Charlotte Bramwell, Jessica Scott, Jason Hancock, Chrysanthi Papoutsi, Mark Pearson, Geoff Wong, Karen Mattick

**Affiliations:** 1 Department of Health & Community Sciences, Faculty of Health and Life Sciences, University of Exeter, Exeter, UK; 2 Department of Public Health and Sport Sciences, Faculty of Health and Life Sciences, University of Exeter, Exeter, UK; 3 Devon Partnership NHS Trust, Exeter, UK; 4 Nuffield Department of Primary Care Health Sciences, University of Oxford, Oxford, UK; 5 Institute of Clinical & Applied Health Research, Hull York Medical School, University of Hull, Hull, UK

**Keywords:** Wellbeing, Typology, Doctors, Mental health, Mental ill-health, Burnout, Intervention, Stress, Resilience, Prevention

## Abstract

**Background:**

The problem of mental ill-health in doctors is complex, accentuated by the COVID-19 pandemic, and impacts on healthcare provision and broader organisational performance. There are many interventions to address the problem but currently no systematic way to categorise them, which makes it hard to describe and compare interventions. As a result, implementation tends to be unfocussed and fall short of the standards developed for implementing complex healthcare interventions. This study aims to develop: 1) a conceptual typology of workplace mental health and wellbeing interventions and 2) a mapping tool to apply the typology within research and practice.

**Methods:**

Typology development was based on iterative cycles of analysis of published and in-practice interventions, incorporation of relevant theories and frameworks, and team and stakeholder group discussions.

**Results:**

The newly developed typology and mapping tool enable interventions to be conceptualised and/or mapped into different categories, for example whether they are designed to be largely preventative (by either improving the workplace or increasing personal resources) or to resolve problems after they have arisen. Interventions may be mapped across more than one category to reflect the nuance and complexity in many mental health and wellbeing interventions. Mapping of interventions indicated that most publications have not clarified their underlying assumptions about what causes outcomes or the theoretical basis for the intervention.

**Conclusion:**

The conceptual typology and mapping tool aims to raise the quality of future research and promote clear thinking about the nature and purpose of interventions, In doing so it aims to support future research and practice in planning interventions to improve the mental health and wellbeing of doctors.

**Supplementary Information:**

The online version contains supplementary material available at 10.1186/s12913-024-10884-6.

## Background

Quality of care in healthcare services is significantly affected by the capacity of the healthcare workforce to deliver it. A key contributor to this capacity is the wellbeing of staff, however, medicine—and healthcare more broadly—is in the midst of a wellness crisis, with high levels of stress, burnout, compassion fatigue, and mental ill-health [1, 2]. This was already urgent before the COVID-19 pandemic, resulting in doctors working when unwell, taking time off or leaving the profession altogether [3, 4], all of which ultimately impact on patient care [5]. These problems have been exacerbated by the COVID-19 pandemic, with increased pressures placed on already strained staff [6–8]. The implications of these issues are widespread, negatively affecting individual doctors, organisations, and patients [1, 3, 9, 10].

Despite the prevalence of these concerns, existing solutions appear to have limited effects, as the situation continues to worsen [11–13]. We have identified two critical gaps in the current approaches to mental health and wellbeing interventions research. Firstly, whilst there is widespread acknowledgement that the causes of the problems are multi-faceted [5, 14, 15], interventions have tended to focus on personal resilience, often aiming to increase the individual’s capacity to manage the demands of their work [10, 16]. Given the complexity and scale of the problem, meaningful interventions need to target multiple aspects of healthcare systems, not individuals alone [10, 15, 16]. As combinations of interventions are commonplace in workplaces, interacting to produce their impacts, we need a way to map multiple interventions in their specific contexts rather than studying them in isolation.

Secondly, there is limited understanding and a lack of specific guidance on how to implement mental health and wellbeing interventions and improve existing offerings [15]. Whilst an array of potential interventions have been suggested, there is not a clear organising framework through which these can be categorised and understood [15]. This is problematic because it poses a barrier to describing and comparing interventions and means that implementation tends to be ill-considered, haphazard, and not tailored to the specific issue. This can result in a plethora of new initiatives, rather than an optimisation of those already in place. The importance of identifying underlying processes and functions within the design and implementation of complex interventions is highlighted within the Medical Research Council (MRC) framework and guidance [17] where it is regarded as essential both for standardisation of intervention design and flexibility within delivery.

Therefore, further work is needed to conceptualise the types of interventions that can be used to support doctors’ mental health and wellbeing at work, and also to develop a way to capture the combinations of interventions offered in practice.

### Study approach

This study drew on the principles of realism to consider the theoretical basis of different interventions by considering the causal mechanisms underpinning them [18]. Realist approaches seek to understand whether programmes (interventions) work, for whom and under what circumstances, how and why. They develop explanatory theory about how outcomes are caused when underlying mechanisms are activated in conducive contexts [18, 19]. Pawson proposed the idea of ‘reusable conceptual platforms’, meaning that interventions—even those with different names and labels, or in different settings—often share common mechanisms, so identifying these groups or ‘families’ of interventions can help identify how and why they work (or not) [19]. This was reflected in our previous research, which found interventions helping to reduce doctors’ mental ill-health at work were based on a limited number of assumptions, such as a supportive workforce culture [15]. In other words, interventions focused on developing policies and interventions that produce a supportive workforce culture could be one ‘reusable conceptual platform’ or ‘family’ of interventions [18, 19].

In this study we develop a conceptual typology by identifying these ‘families’ of interventions that could be used to support doctors’ mental health and wellbeing at work, and to also create a mapping tool through which the different interventions offered in practice can be mapped and described. We anticipate that the conceptual typology and mapping tool will help those researching and implementing interventions to clarify their nature and purpose, and in doing so use them more effectively to improve the mental health and wellbeing of doctors.

### Use of terms

We have taken a broad and inclusive approach, reflecting the reality of practice in medical organisations, using the term “mental health and wellbeing” to encompass a wide range of intended outcomes. We recognised the challenges of defining terms such as “wellbeing” and “mental health”, not least because these are sometimes included in the same definition and other times considered separately. We also wanted to ensure that our study included a spectrum of interventions from those designed to proactively support the mental health and wellbeing of the whole workforce, to those targeting individual doctors with specific problems.

Similarly, "intervention" was also defined broadly: as any action, activity and/or resource designed to improve doctor wellbeing and/or mental health. This included interventions designed to operate at different levels, including individual, team, organisational, and national.

## Methods

### Aim

To conceptualise and categorise existing workplace mental health and wellbeing interventions for doctors.

### Objectives

To develop:

1. a conceptual typology of workplace mental health and wellbeing interventions.
2. a mapping tool to apply the typology within research and practice.

### Study design

The study was an evidence synthesis of peer-reviewed literature and existing practice-based interventions, drawing on existing theories and gaining stakeholders’ insights. The development of the conceptual typology and the mapping tool were concurrent and involved multiple iterations.

### Involving stakeholders

Given the messy reality of healthcare practice, proposed solutions are most likely to be successful if researchers co-create them with practitioners [17, 20]. Doing so draws on the implicit local and experiential knowledge of a range of relevant stakeholders in a way that acknowledges the full complexity of healthcare environments, policies, and processes. Therefore, the study was supported by an online stakeholder group, drawn from multiple healthcare settings across England, which met regularly to discuss the research process, findings, outputs, and dissemination. The stakeholders represented different perspectives, including doctors from shortage specialties, doctors who have experienced mental ill-health, other healthcare professionals and managers, patients and the public, and doctor support organisations.

### Locating interventions for typology development

We started by identifying interventions within the 179 included papers from our recent ‘Care Under Pressure’ (CUP1) realist review on the same topic [15]. Details of the 179 papers are available in Table 2 of the published report [15]. As we wanted to ensure that the typology was inclusive of the full range of interventions available, we considered the full set of papers from this review. These reported on both existing and recommended interventions, and also included expert opinion and policy reports in addition to published research studies. Examples of interventions in this literature included mindfulness training [21], Schwartz rounds [22], and wellness programs [23].

**Fig. 1:**
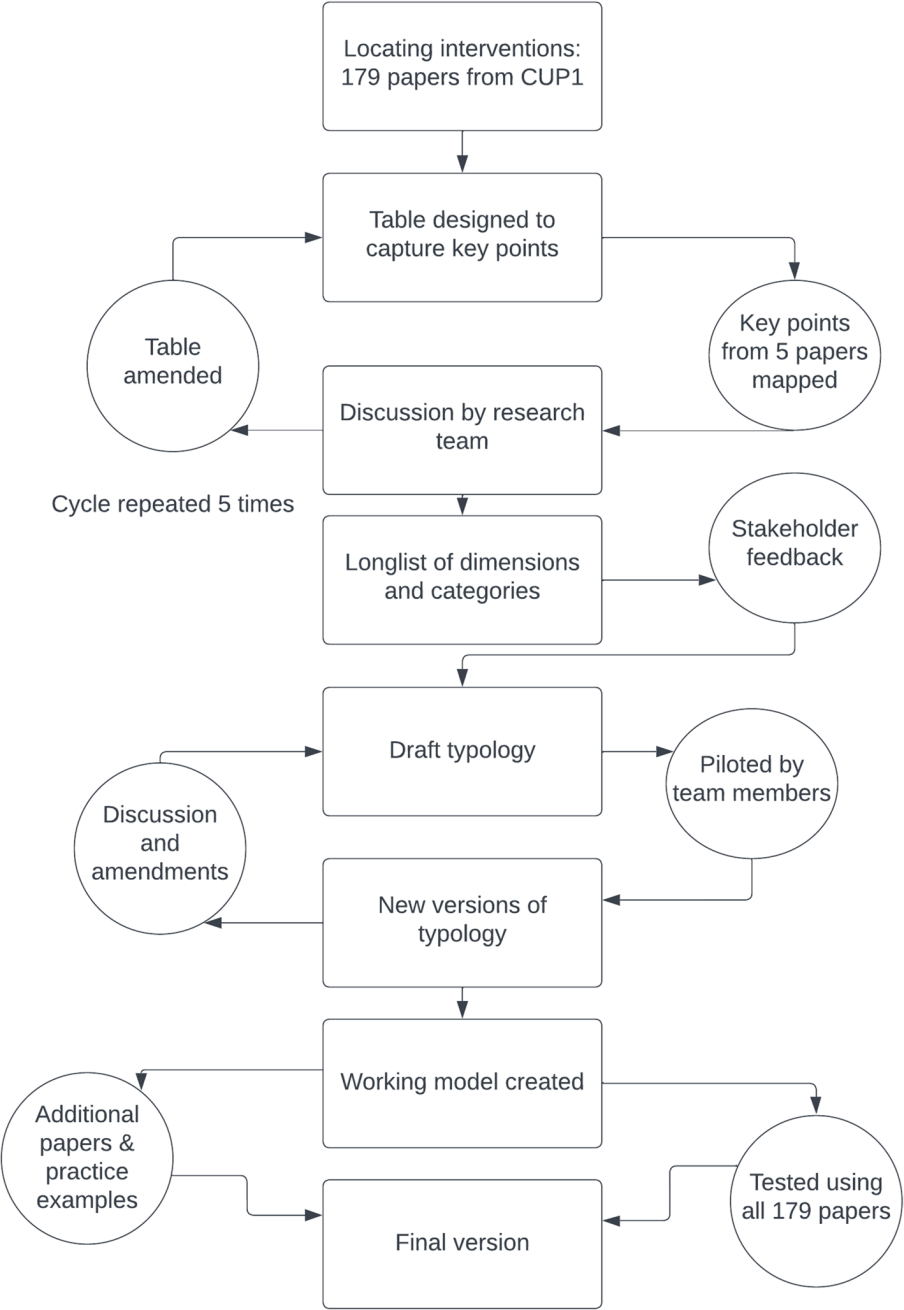
The process of typology development and testing

The full process of typology development and testing is shown in Fig. 1.

### Analysing interventions and developing the initial typology

An initial subset of the 179 articles was reviewed to start identifying potential types of interventions. Five intervention papers were selected with diverse methodology (quantitative, qualitative, mixed) and intervention level (individual, structural, mixed). An Excel spreadsheet was designed to capture key points, including the targeted problem, mechanism, duration, and who was responsible for implementation. Data was extracted from the papers concurrently by five authors (AM, AP, CB, DC, KM). After discussion, the table was amended and a further five papers were selected on the same basis and analysed, by six authors (AM, AP, CB, DC, KM, JS).

A log of the amendments and reflections on challenges with the process was kept for discussion at weekly typology development meetings. This process continued until 25 papers had been reviewed. We explored different ways of structuring the typology, including using dimensions along a continuum (e.g. prevention-treatment; accessible-hard to access; informal-formal) and categories (e.g. helping people to cope/ making people feel valued). The work was shared with the full research team for comment and discussion, and a ‘long list’ of possible dimensions and categories was developed and shared with the stakeholders for feedback.

### Further iterations of the typology

From the stakeholder feedback, we narrowed the initial longlist into a shortlist of dimensions and categories, which were used to pilot possible typology options. This process identified that the published literature did not typically include sufficient information to plot interventions confidently on a continuum of any of the identified dimensions, so we decided to focus on using categories instead. Categories were developed through an iterative combination of analysis of published interventions, application of relevant theoretical concepts, and team dialogue. For example, we explored classifications from the stress-reduction literature [24, 25], reviewed the CUP1 programme theory [15] and considered categories which had been proposed in a previous systematic review [26]. We subsequently trialled further versions of the typology, with categories based on the level of intervention (primary/secondary/tertiary [25, 27]), type of intervention (descriptive subcategories), and purpose of the intervention (e.g. whether an intervention aims to remove a negative aspect or improve a positive one [28]). This latter point is a key concept from positive psychology, described as choosing between a red cape and a green cape by Pawelski [28], and refers to whether the focus is to grow desirable things or reduce undesirable things. As Pawelski explains: “*the positive is not the same thing as the absence of the negative; well-being is not the same as the absence of ill-being*” (p.361). Piloting of the draft typology at this stage revealed little convergence between researchers, reflecting issues of clarity in the literature leading to different interpretations. For example, published interventions often reported multiple purposes (e.g. reduce stress and increase satisfaction) or the intentions were hard to assess (e.g. was a discussion group intended to improve connection, provide support or increase learning?).

Multiple research team members (AM, AP, CB, KM) independently created new versions of the typology, based on our experiences so far. To supplement the typology work created through the published literature, we trialled some practical examples of real interventions from NHS hospitals. We exchanged thoughts via a shared document, which enabled a detailed ongoing discussion. Via this process, we reached consensus on a working model, which proved effective with both literature and practice-based examples. Definitions of each category and subcategory were developed to provide clarity and support future users of the tool. An “other” column was also included within each subsection to identify any other possible categories. The draft conceptual typology and mapping tool was shared with the stakeholder group, leading to further minor amendments.

### Applying the mapping tool to published interventions

Having developed a draft mapping tool, this was applied to all 179 included papers from the CUP1 realist review [15] by three co-authors (AP, AM, CB), with regular meetings with the wider team to discuss any differences in mapping and papers that proved difficult to categorise. Based on published literature and conversations with experts and stakeholders, we anticipated that COVID-19 had changed the extent and profile of mental ill-health and the associated interventions, but we did not anticipate that COVID-19 had changed the fundamental nature of these. To test our assumptions we identified those papers which had cited the CUP1 realist review, four of which had described an intervention [29–32]. We identified that these four research articles could also be mapped using the draft mapping tool. Having completed the mapping exercise, we asked the stakeholder group to review the typology wording at this final stage, to ensure it was clear and relevant for the intended users.

### Reflexivity

We considered reflexivity to be an integral part of the full research process [33] and built in regular opportunities for reflection and discussion. To ensure the research benefited from diverse perspectives, the research team represented multiple disciplines, professions, career stages, and nationalities, including doctors who are currently navigating the system and academics who have studied it. We met regularly throughout the study to share literature, question each other’s interpretations, build consensus to agree the typology and reflect on how the typology might be used.

## Results

The objectives of this study were to develop both a conceptual typology of workplace mental health and wellbeing interventions, and a mapping tool which could be used to apply the typology within both research and practice. In total, 183 papers were used to develop and test the conceptual typology and mapping tool. Whilst both were developed concurrently, we have presented these separately for clarity.

### Conceptual typology

**Fig. 2:**
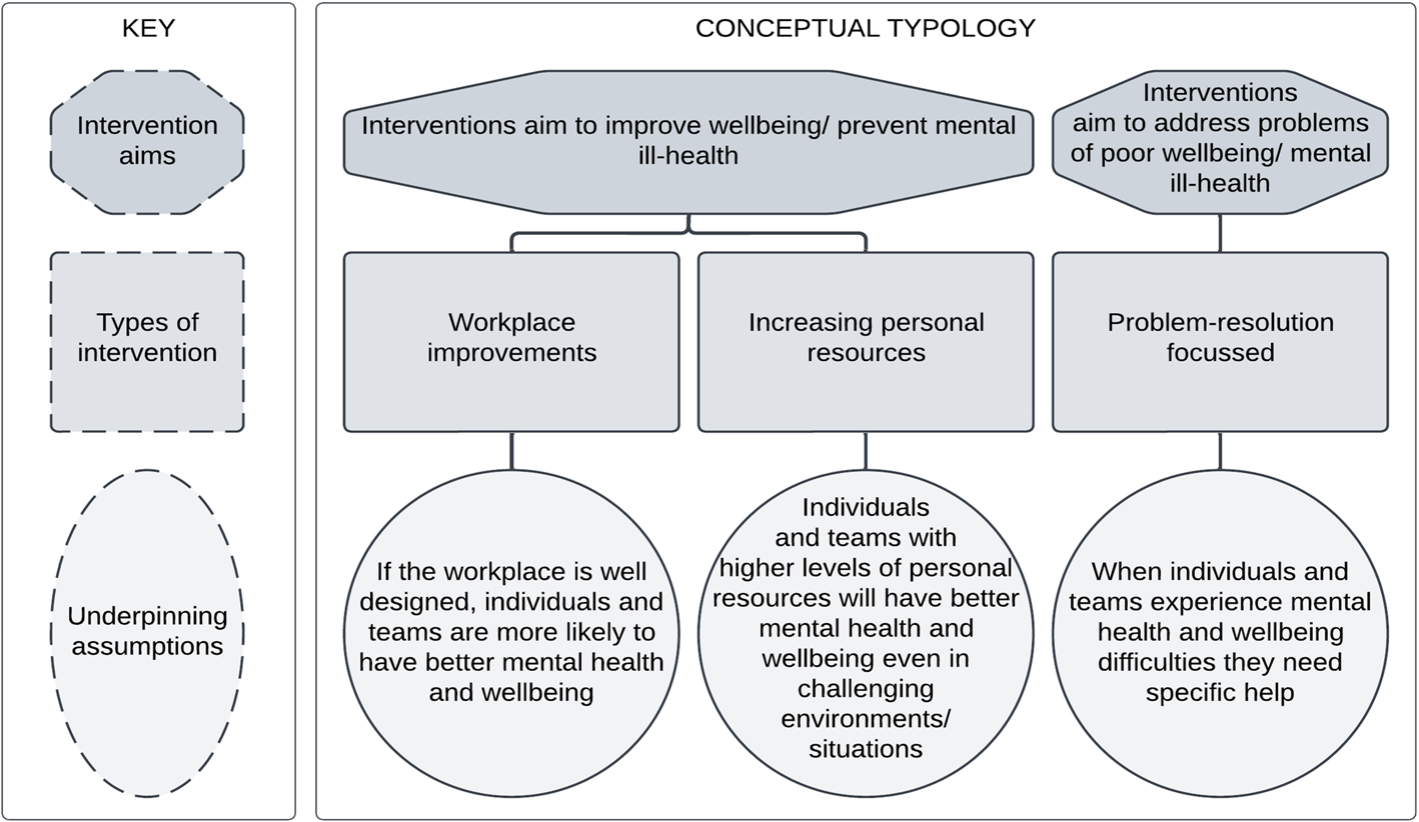
Conceptual typology of mental health and wellbeing interventions

Our conceptual typology (shown in Fig. 2) divides mental health and wellbeing interventions into three fundamental categories. These are based on whether the intervention was preventative at a systemic level through workplace improvements, preventative at an individual level through increasing personal resources, or problem-resolution focussed. Whilst this categorisation has some synergies with stress reduction literature [25] there are also some key differences which help to clarify the focus and purpose of the intervention. Categorising interventions which are preventative and involve workplace changes builds on the idea of primary types of stress reduction interventions [27] in that they are designed to be preventative. However, they differ from this definition in that they might not necessarily be proactive. For example, improvements to rota processes or to peer support might happen in response to a challenge or difficulty within the workplace, rather than as planned provision. This category also relates to the systems perspective of resilience [34]: where the environment is regarded as a crucial element in both supporting and interacting with individuals. Whilst much of the literature refers to individuals, we have extended our underpinning assumptions to also incorporate teams, given that some interventions are based on team working.

Those interventions which are preventative and based on improving personal resources include all those activities and resources designed to strengthen the ability of the individual, or team (and therefore the organisation as a whole), to handle the challenges of the workplace. These all included elements of information and/or training as one of the primary ways identified of strengthening personal resources. This section has theoretical links to both the primary and secondary categories of stress reduction [25, 27] in that interventions included are designed to be preventative (in line with primary level stress interventions) and are also designed to support individuals’ (or teams’) responses to stress (in line with secondary stress management interventions). This section of the conceptual typology also draws on aspects of resilience and training literature, whereby developing an individual’s knowledge, skills and aptitudes are believed to improve both self-efficacy and compassion-satisfaction [35, 36].

The categorisation of problem-resolution focussed interventions includes all those designed to solve a mental health or wellbeing problem after it had arisen. This has close alignment with the “tertiary” categories of stress management interventions [25, 27], but extends the solutions to those for physical health and non-work problems in order to encompass the full range of reported interventions.

It is important to note, however, that some interventions included elements across multiple categories (e.g. both workplace improvement and strengthening personal resources). Each category also included multiple sub-categories of intervention, and the developed mapping tool (see below) enables more granular mapping to reflect the nuance and complexity of the interventions reviewed.

### The mapping tool

Where the conceptual typology enables interventions to be considered in terms of their fundamental theoretical assumptions, the mapping tool was designed as a practical tool to support both practice and future research.

**Fig. 3:**
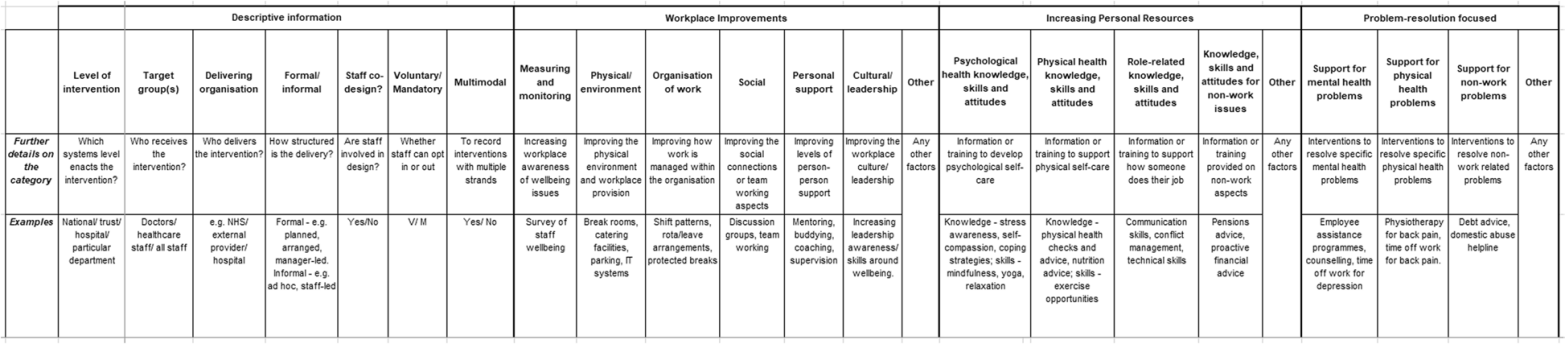
Mapping tool for mental health and wellbeing interventions

The mapping tool is shown in Fig. 3, and seven worked examples are included within Additional file 1. It includes a descriptive section plus three further sections which categorise the intervention in line with the conceptual typology categories (workplace improvements, increasing personal resources, and problem-resolution focussed), and then includes subcategories to enable the intervention to be fully mapped and described. Each section also includes an “other” subcategory, to enable any aspect of interventions which does not easily fit in another subcategory to be included.

The descriptive information section encompasses aspects of the way the intervention was created and established. These do not pertain to the mechanism of the intervention, but to features regarding its design and implementation which are still likely to be relevant in the way that an intervention is perceived. This section is completed for all interventions, and then the intervention characteristics are mapped across the remaining sections.

**Fig. 4:**
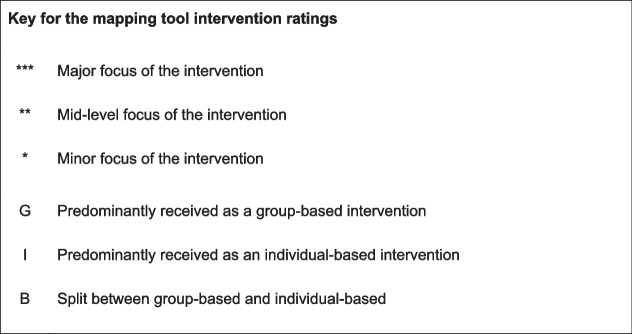
Mapping tool key

It became evident that many reported interventions did not completely sit within just one category of the tool. It is therefore designed such that interventions can be mapped across multiple categories: for example to show that one intervention primarily fits within one category but includes elements of others. This is achieved through the use of a “*” system (see Fig. 4). In using this approach, each intervention can be mapped into multiple categories whilst still identifying its major focus.

The secondary element of the mapping process is to indicate if the intervention is delivered on a group or individual basis, or if it had elements of both (Fig. 4). This enables the format of delivery of each element of the intervention to be mapped as well as the intervention’s intended purpose.

## Discussion

The aim of this research was to develop a conceptual typology and a mapping tool of workplace mental health and wellbeing interventions for future research and practice. This is needed to provide a better oversight of the interventions that have already been researched and implemented [15], and to support better strategic planning of further research and practice. This also supports future research investigating mental health and wellbeing interventions in identifying the underlying processes, in turn enabling better alignment with MRC implementation guidance for complex interventions [17].

The term “typology” can span a wide range of mapping and categorising exercises, and most of these aim to simplify characteristics into a very small number of types (for example [37–39]). However, given the complexity of mental health and wellbeing, it was perhaps not surprising that interventions did not readily map into a limited number of easily defined categories. Whilst it is suggested that typology development often involves a two-step conceptual-empirical approach [37], in our case there were instead multiple iterations and refinements. These were based both on the features of identified interventions and consideration of theory, relating to both stress and burnout, and positive psychology (e.g. wellness and resilience). In doing so our work extends and connects some of the previous research frameworks in these areas. For example, it draws from the primary, secondary and tertiary intervention distinction in the stress prevention literature [25] in that it identifies two levels of prevention and one of problem-resolution. It then differs from this approach in the differentiation between the preventative categories by building from aspects of resilience literature [34] based on whether actions aim to improve the environment (workplace systems level) or strengthen the individual and/or team.

As a result of the multiple iterations and developments, the mapping tool created has a level of detail which enables the nuance and complexity of the interventions to support doctor mental health and wellbeing to be mapped. The mapping tool created in this study was comprehensively tested with existing research and practice examples, and each of the interventions described could be mapped into it. Many of the interventions mapped to at least two of the subcategories, demonstrating the value of this new mapping tool for mapping complex and multi-faceted interventions, rather than restricting these into one category.

The typology development process itself was not straightforward. As well as the complexity of the topic in question, the other main challenge in creating the conceptual typology (and then utilising the associated mapping tool) was the limited consistency in terminology within the papers reviewed. It was also sometimes difficult to establish the theoretical basis for the intervention: for example, which particular aspect of wellbeing the intervention aimed to improve, why the intervention chosen might be the best fit, and what it was about the particular intervention that was designed to be particularly useful to participants. For example, we initially tried to use the red cape/ green cape principles [28], which suggest that interventions can be categorised based on whether they aim to reduce the negative aspects of work (to reduce burnout and stress) or to strengthen the positive aspects (to improve job satisfaction). However, we found that many papers reported that the intervention aimed to do both and so this categorisation was not helpful. With input from realist methodologists on our team, we attempted to determine the underlying assumptions (mechanisms) of the intervention, but not all papers were clear or provided sufficient details. Furthermore, mapping interventions also highlighted the different ways in which interventions of the same name are used. For example, some Balint groups placed the emphasis on the importance of group support, whilst others focussed on their importance for role-related learning. Likewise, counselling was often used as a problem-resolution approach for someone with identified mental health needs, but was also used proactively in others as a way of strengthening personal resources.

Whilst not part of the original aim, in addition to developing the typology and the tool, completing the mapping exercise for the selected papers also enabled some initial observations to be made about the interventions reported in the literature to date, which may indicate areas for future research in this area.

The first of these was that there were far fewer interventions reported that targeted workplace improvements compared to those designed to strengthen personal resources. This could be explained by the relative complexity of some of these: changing the culture is arguably much harder to implement and research than the addition of a training course. However, this may also suggest that the thinking behind the interventions is that the workplace does not need to change but rather the individuals within it: this relates back to conceptualising resilience as an individual trait or characteristic rather than the more systems-based thinking that is developing in the wider literature [34]. The problem of whom is responsible for wellbeing has been raised by others, such as Jackson et al., [40] who argue that the “neoliberal transformation of well-being” (p.4) has placed the responsibility with the individual to keep themselves well, rather than with the organisation to create the conditions needed for people to be well.

It was also evident that very few of the interventions included input from healthcare staff (the intended end users) in their design, or in the choice of intervention being introduced. Where staff were involved in co-design at all this was typically in relation to relatively minor elements (e.g. which topics were chosen for a discussion group), rather than in more structural elements of the intervention. Given that feelings concerning autonomy may in themselves impact on job satisfaction [41], and also that the success of a change being adopted might be affected by levels of staff engagement [42], the way in which staff are involved in intervention implementation may also merit further investigation.

Another area that might be signalled for future research is the low number of group-based interventions in the problem-resolution section. By noting whether interventions were implemented on a group or individual basis we noticed that most of the personal resource building interventions were implemented on a group or mixed basis. In contrast, almost all of the problem-resolution interventions were delivered through a one-to-one approach. Whilst acknowledging that aspects of confidentiality might deter some medical professionals from seeking a group-based solution, the benefit of peer group support has been well documented in other areas of mental health recovery [43, 44], and it would appear to be a gap within the current research relating to doctor mental health interventions.

It also became evident that few interventions included a physical element: either the physical nature of the workplace, in the strengthening of individuals’ physical health or in solving physical health issues. This is in part explainable by the fact that the initial literature search was for mental health and wellbeing interventions: it does, however indicate that there was a separation between physical and mental health in the interventions designed or recommended. Whilst the literature recognises the impact of physical health interventions on psychological health in the wider population [45] and in other workplaces [46] it would appear that when mental health interventions are designed for doctors these principles are not as typically included. Investigating the effects of physical health interventions on mental health and wellbeing for doctors may also be a useful area for future research.

Given the challenges discussed earlier, a key recommendation when future research is both undertaken and published will be for researchers to make clear their rationales for the selection of a specific intervention and to incorporate a greater level of detail both about the intervention itself and its underpinning mechanisms. This supports the principles of checklists have been developed for interventions such as the “template for intervention description and replication” (TIDieR) checklist [47], but also extends these recommendations by highlighting the need for a clear articulation of the underpinning mechanism and assumptions. The typology may be helpful for future research teams to map the key aspects of their study, and to ensure these elements are included within their publications. Aligned to this, it would also be beneficial for future studies to clearly define both the intervention and any terminology used, to support both those receiving or using the intervention and those researching it.

### Strengths and limitations

A strength of the work is the range of papers included in the development process, which included peer-reviewed academic publications and also healthcare reports and practitioner commentaries. The involvement of multiple researchers with different disciplinary backgrounds, together with feedback from a diverse range of stakeholders, has meant that there have been multiple iterations of the typology and the tool with each development adding a new perspective. This approach has helped to shape a mapping tool that can be used to map interventions to reflect their complexity and nuance which in turn enables further patterns to be identified. For example, the use of a “*” system to indicate the major/minor focus of an intervention helps to indicate those with a clear focus and where a more generalised approach is taken. The identification of whether interventions are delivered in a group or individual basis enables potential gaps and further questions to be considered: for example, if there would be any benefit in using a group-based approach to problem-resolution interventions.

Reviewing the literature relating to typology creation, it became apparent that few papers clearly articulate their approach. Due to this, we have attempted to articulate the detail underpinning the typology development, not just for the purposes of transparency but to also provide methodological insights for others seeking to develop typologies.

As with all research studies, there were also some limitations to our approach. As well as a strength in terms of the range of interventions reviewed, the use of an extensive range of papers is also a limitation of the typology, in that it is not a map of “what works” as the interventions used to inform its development include *all* those reported, whether they had an effect or not, and whether they have been implemented or are just at the recommendation stage. This limits the extent to which any of the observations of the interventions themselves could be reported at this stage beyond those identified as noticeable gaps. However, even with this limitation it was evident that some types of intervention were rarely reported: for example, the low number of physical interventions in any section, and the dearth of group-based interventions for those with mental health difficulties.

A further limitation is that only four of the papers that we used to test the typology and mapping tool have been published since 2020, because our experts and stakeholders recommended that we prioritise our limited resources into trialling some practical examples of real interventions from NHS hospitals. Finally, given the constraints of health service budget, it is acknowledged that cost comparisons between interventions would be helpful. This was not, however, an aspect typically reported within the literature reviewed relating to health and wellbeing interventions and therefore not included within this paper.

## Conclusion

The creation of this conceptual typology and mapping tool has implications for other researchers, policy, and practice. For researchers it may support the theoretical understanding of the purpose of an intervention, and also enable planned interventions to be mapped out. In doing so it may help to clarify this purpose: for example, which specific aspect(s) it is designed to target beyond general aspirations of improving wellbeing or reducing burnout. It may also provide a framework for the information that could usefully be included within any research publications, or for any practice-based recommendations.

The mapping tool has important potential use for policy-makers, and for senior leaders looking to assess or implement wellbeing interventions in practice. In both cases this enables existing interventions to be mapped against the framework, and to identify gaps at a workplace, local, or national level. Using the mapping tool may also help to clarify the benefit of any newly proposed interventions: for example, whether they are designed to improve the workplace, increase personal resources, and/or resolve problems after they have arisen. The mapping tool could also potentially be used in reverse: to map where the problems are within an existing healthcare organisation and use this as a basis for planning improvements.

### Implications for further research

Future research that would add value to this area of work could be to use the mapping tool to map only those interventions that have been identified as having a positive impact on the mental health and/or wellbeing of participants. This may help to create a useful overview of the features of existing interventions that have been identified as being beneficial, whilst also identifying areas where further research may be valuable.

### Supplementary Information

**Additional file 1.** Mapping tool with worked examples.

## Data Availability

The secondary data which was used to develop the typology and mapping tool is located within Carrieri D, Pearson M, Mattick K, Papoutsi C, Briscoe S, Wong G & Jackson M. Interventions to minimise doctors' mental ill-health and its impacts on the workforce and patient care: the Care Under Pressure realist review. *Health Serv Deliv Res* 2020;8(19). https://doi.org/10.3310/hsdr08190.

## Abbreviations

CUP1: Care under Pressure 1
MRC: Medical Research Council
TIDieR: Template for intervention description and replication
